# Use of antibiotics contrary to guidelines for children’s lower respiratory tract infections in different health care settings

**DOI:** 10.1007/s00431-023-05099-6

**Published:** 2023-07-19

**Authors:** Roope Poutanen, Matti Korppi, Peter Csonka, Satu-Liisa Pauniaho, Marjo Renko, Sauli Palmu

**Affiliations:** 1 Center for Child, Adolescent and Maternal Health Research, Tampere University and Department of Pediatrics, Tampere University Hospital, Tampre, Finland; 2 Faculty of Medicine and Health Technology, Tampere University, Tampere, Finland; 3 Terveystalo Healthcare, Tampere, Finland; 4 Department of Paediatrics, Kuopio University Hospital, University of Eastern Finland, Kuopio, Finland

**Keywords:** Antibiotics, Care guidelines, Community-acquired pneumonia, Lower respiratory tract infections

## Abstract

This study aimed to evaluate antibiotic prescriptions for children with lower respiratory tract infection (LRTI) in public and private primary care clinics and in a hospital’s pediatric emergency department (PED) in 2012–2013 (pre-guideline) and in 2014–2015 (post-guideline). Special attention was paid to guideline compliance, especially regarding macrolide prescriptions, which the guidelines discourage. Retrospective data of 1431 children with LRTI in November–December 2012–2015 were collected from electronic registers and checked manually. Three diagnostic groups were analyzed: community-acquired pneumonia (CAP), wheezing bronchitis, and non-wheezing bronchitis. A comparison of the pre- and post-guideline periods revealed antibiotic prescription rates of 48.7% and 48.9% (*p* = 0.955) for all LRTIs, respectively, and 77.6% and 71.0% (*p* = 0.053) for non-wheezing bronchitis. The prescription rates for all LRTIs were 24.9% in PED and 45.9% in public (*p* < 0.001 vs. PED) and 75.4% in private clinics (*p* < 0.001 vs. PED and *p* < 0.001 vs. public clinics). During post-guideline periods, antibiotics were prescribed for CAP less often in private (56.3%) than in public clinics (84.6%; *p* = 0.037) or in PED (94.3%; *p* < 0.001 vs. private and *p* = 0.091 vs. public primary clinics). Macrolide prescriptions were highest in private clinics (42.8%), followed by public primary care clinics (28.5%; *p* < 0.05) and PED (0.8%; *p* < 0.05 vs. both public and private primary care). Amoxicillin was the predominant antibiotic in public primary care and PED and macrolides in private primary care.

*Conclusion*: Antibiotic prescribing for children with LRTI differed significantly between healthcare providers. CAP was undertreated and bronchitis overtreated with antibiotics in primary care, especially in the private clinics.

**Table:** 

**What is Known:** *• Clinical Treatment Guidelines tend to have modest effect on physicians’ antibiotic prescribing habits.* *• Pediatric viral LRTIs are widely treated with unnecessary antibiotics.*	
**What is New:** *• Remarkable differences in antibiotic prescriptions in pediatric LRTIs between Finnish private and public providers were observed.* *• Overuse of macrolides was common especially in private clinics.*

**Supplementary Information:**

The online version contains supplementary material available at 10.1007/s00431-023-05099-6.

## Introduction

Lower respiratory tract infections (LRTIs) in children can be divided into bronchiolitis, bronchitis, and pneumonia, among others. Bronchiolitis and bronchitis are caused by respiratory viruses such as respiratory syncytial and rhinoviruses. Bronchiolitis affects small airways, occurs in infants, and has crackles or wheezing as typical clinical findings [1]. Bronchitis affects both small and large airways and may present with or without wheezing [1].

The diagnosis of community-acquired pneumonia (CAP) is clinical and radiological or laboratory studies are not needed in uncomplicated cases [2–4]. However, similar symptoms and signs, such as cough, fever, tachypnea, and crackles on auscultation, occur in children with other LRTIs [1, 5]. There are substantial variations in how physicians interpret clinical findings and diagnose different LRTIs [1].

Viral bronchiolitis and bronchitis are self-improving infections, and antibiotics are not indicated [5]. The current evidence-based guidelines recommend that children with CAP should be treated with amoxicillin or narrow-spectrum penicillin, which are effective against *Streptococcus pneumoniae* [2–4]*.* Macrolides are not recommended for monotherapy because *S. pneumoniae* is not susceptible enough [2, 4].

Antibiotic overconsumption in respiratory tract infections belongs to the most important healthcare problems in European children [6]. Reduced consumption and more targeted prescriptions are necessary to decrease the emergence of resistant bacteria.

The current study evaluated antibiotic prescriptions for Finnish children with LRTIs in public and private primary care clinics and in the pediatric emergency department (PED) of an academic hospital in 2012–2015. Special attention was paid to how well doctors complied with the Finnish Current Care Guidelines for children’s LRTIs, specifically regarding the prescribing of macrolides, which is discouraged by such guidelines. The Guideline was published in June 2014 in Finnish language and is freely available online in Finnish Current Care database [4, 5]. The methods for implementation of the guidelines were lectures in professional meetings and summaries in medical and pharmaceutical journals. The effect of implementation was not systematically measured.

## Materials and methods

We collected data on pediatric visits for LRTI during the infection seasons in 2012–2015 from the electronic registers of Tampere’s public primary care clinics, Tampere University Hospital’s PED, and two private primary care clinics of Terveystalo. After the electronic identification of cases, the registered data were manually checked by two authors (RP and SP).

Tampere is a city with 240,000 inhabitants (34,000 children < 16 years old) in Southwest Finland, and Kouvola a town with 80,000 inhabitants (11,000) in Southeast Finland. Terveystalo, the largest private healthcare company in Finland with over 300 clinics across the country, has clinics in Tampere and Kouvola.

### Attending healthcare clinics

During the study years 2012–2015, the city of Tampere (Tampere Health Center) provided primary public healthcare at 13 stations that shared an electronic patient register and served patients, including children with acute infections, during office hours. Both adults and children were treated by general practitioners (GPs).

In addition, Tampere Health Center provided primary emergency care round-the-clock at the emergency room (ER), which was responsible for emergency care of 62,000 children aged 0–15 years living in the city and nearby areas. Children were treated by GPs or residents in general or emergency medicine. The ER doctors could consult by phone the hospital’s pediatricians if needed. Children who visited the ER were either discharged to home or admitted to hospital’s PED.

Tampere University Hospital’s PED provided secondary care for a population of 90,000 children aged 0–15 years. Patients from public or private primary care clinics in the region were admitted therein. There was a substantial overlap between child populations for which public primary care clinics, the healthcare center’s ER, or the PED were responsible.

The private clinics in Tampere and Kouvola reported 11,500 and 6100 annual visits on average in 2012–2015 for children aged 0–15 years, respectively. The children were treated by pediatricians, ear, nose, and throat doctors, or GPs, and rarely by other doctors.

We classified the attending healthcare units into three categories. Tampere University Hospital’s PED represented secondary healthcare. The healthcare centers’ ER and stations represented public primary care, and the two private clinics represented private primary care.

### Data collection

Tampere healthcare center’s stations and ER used the same register, and their data were analyzed in combination. The private clinics used Terveystalo’s nationwide register, and again, their data were analyzed in combination. The PED used the University Hospital District’s register. We collected data from children aged 0–15 years who had visited attending units during November and December in 2012–2015. These months were chosen as they represent high infection rates within the infection seasons. The study periods in 2012–2013 represented the era before the Current Care Guidelines for children’s LRTIs in July 2014 and the study periods in 2014–2015 the era after.

We included visits with the following International Classification of Diseases 10th Edition (ICD-10) codes: influenza (J11), pneumonia (J18), acute bronchitis (J20), and bronchiolitis (J21). LRTI cases were classified into three diagnostic groups: CAP, non-wheezing bronchitis, and wheezing bronchitis which also included bronchiolitis. In Finnish clinical practice, the code J21.90 is used for wheezing bronchitis and the other J21 codes for bronchiolitis.

We collected data on the ICD-10 codes, visit days, patient’s ages, and the antibiotics that were prescribed. No outcome data were collected. The data was managed as coded. Coding was done separately for public primary, private primary, and secondary care clinics.

### Antibiotic prescriptions

The current study focused on antibiotic prescriptions for children with CAP, non-wheezing bronchitis, and wheezing bronchitis treated at home. We excluded LRTI patients with otitis media because according to the current recommendations, they are usually treated with antibiotics [7]. Since we did not know exactly which patients were transferred to the ward, we excluded those who received intravenous antibiotics. We had no information of antibiotic allergies or previous prescriptions, which might guide antibiotic selection.

### Guideline concordance

The Current Care Guidelines do not recommend antibiotics for bronchitis, which is a self-recovering viral infection [4]. Thus, avoiding antibiotics is a guideline-concordant approach in both wheezing and non-wheezing bronchitis. Instead, for CAP, the Current Care Guidelines recommend antibiotics, which cover *Streptococcus pneumoniae*. Amoxicillin is the first-line antibiotic at all ages, combined with macrolides for > 5-year-olds and doxycycline for > 8-year-olds, if *Mycoplasma pneumoniae* is suspected [4].

### Statistics

The data were managed with SPSS Statistics for Windows, version 26.0. (IBM Corp, New York, USA). The chi-square test and Fisher’s exact test were used for statistical analyses, as appropriate.

### Ethics

This study was based on the registers of the hospital, healthcare center, and private clinics. According to Finnish law, Ethics Committee approval was not needed since the patients were not contacted. The study was carried out with permissions of the chief doctors of Tampere healthcare center, Tampere University Hospital and Suomen Terveystalo.

## Results

### Study populations

**Fig. 1:**
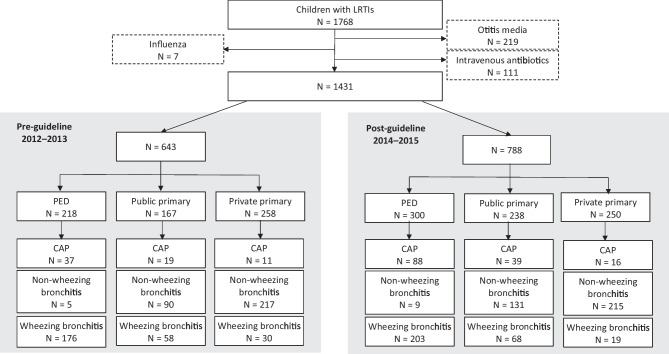
Flowsheet of the study. Numbers of cases in the pediatric emergency department and public and private primary care clinics by the diagnoses of community-acquired pneumonia (CAP) and wheezing or non-wheezing bronchitis presented separately for the pre- and post-guideline study periods

Overall, 1768 children met the LRTI criteria. We excluded 219 children who had otitis media, 111 children who were treated with intravenous antibiotics, and 7 children with influenza. The final study group consisted of 1431 patients (60.5% boys); 643 were treated in 2012–2013 and 788 in 2014–2015 (Fig. 1 and Table S1).

During the whole study period, PED treated 518 children, public primary care clinics 405 (health stations 168, ER 237), and private primary care clinics 508 (Tampere 222, Kouvola 286). Sixty percent of CAP and 68% of wheezing bronchitis cases were treated in PED and 65% of non-wheezing bronchitis cases in private primary care clinics (Fig. 1). The patients treated in PED were younger (median age 1.4 years [IQR 0.6–2.8]) than those treated in public (3.2 [1.5–7.8], *p* < 0.001) or private primary care (4.8 [2.2–9.1], *p* < 0.001). Median age of the total cohort was lower in 2012–2013 (2.5 [1.2–5.8]) than in 2014–2015 (3.1 [1.3–7.3], *p* = 0.05) (Table S1).

### Antibiotic prescriptions

**Fig. 2:**
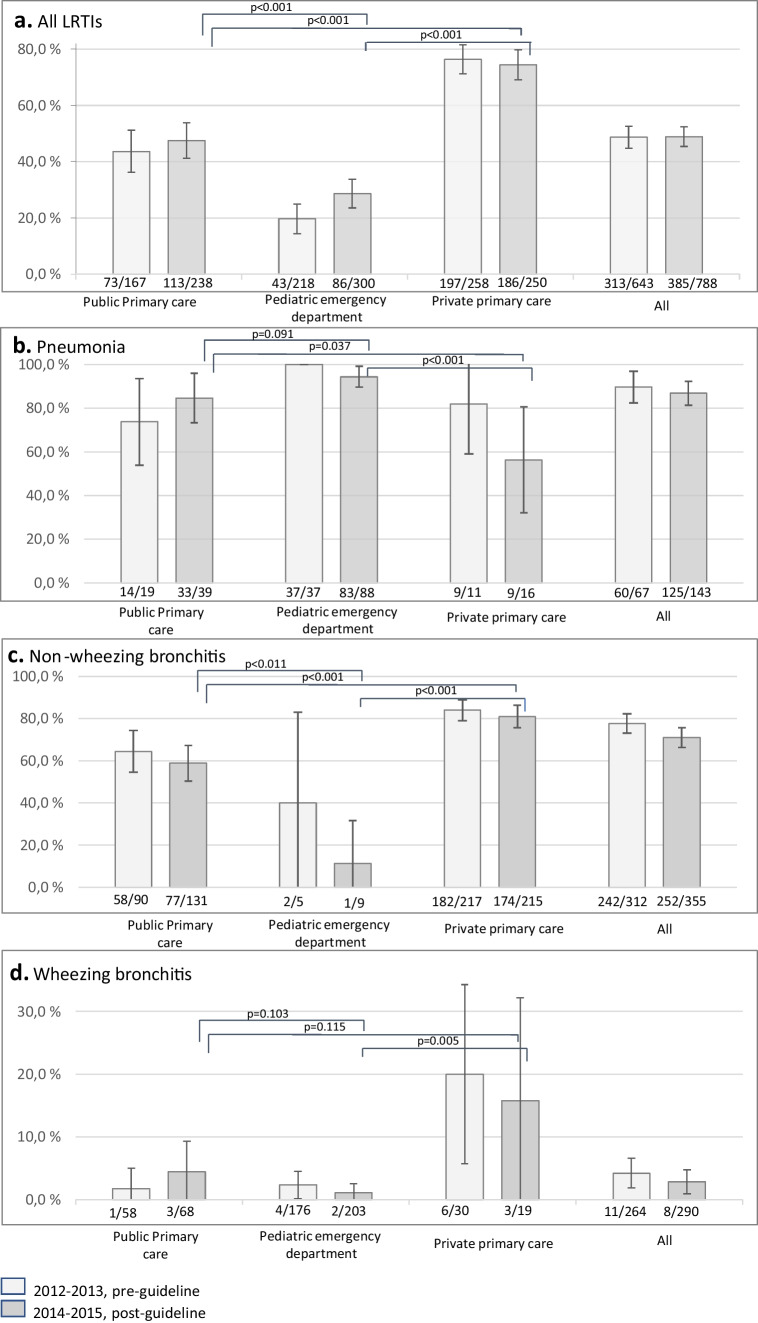
Comparison of the proportions of patients who received antibiotic therapy for (**a**) All LRTIs, (**b**) pneumonia, (**c**) non-wheezing bronchitis, and (**d**) wheezing bronchitis. Comparisons were made between two study periods: 2012–2013 and 2014–2015. The figure displays 95% confidence intervals representing the differences in antibiotic usage before and after the implementation of the guidelines within healthcare providers. Additionally, it includes *p* values to illustrate the difference between providers in the post-guideline period. The bottom row of each subfigure details the ratio of antibiotic prescriptions per patients

In all, 698 visits (48.8%) ended with an antibiotic prescription: amoxicillin in 45.6%, azithromycin in 25.2%, amoxicillin–clavulanic acid in 13.0%, clarithromycin in 5.9%, cephalexin in 3.5%, doxycycline in 2.7%, and others in 4.4%. Two antibiotics (amoxicillin + macrolide) were prescribed for 13 patients only. These cases were included in the amoxicillin group in the analyses. The number of antibiotic prescriptions was 313 (48.7%) during the pre-guideline and 385 (48.9%) the post-guideline study periods (Table S2). The prescription rates were 24.9% in PED and 45.9% in public (*p* < 0.001 vs. PED) and 75.4% in private primary care clinics (*p* < 0.001 vs. PED and *p* < 0.001 vs. public clinics, respectively) (Fig. 2a).

Amoxicillin was the predominant antibiotic in public primary care clinics and PED (Table S2). Macrolides were predominant antibiotics in private primary care clinics (42.6%), followed by public primary care clinics (28.5%; *p* < 0.05) and PED (0.8%; *p* < 0.05 vs. both public and private primary care). In private clinics, the pre- and post-guideline figures for macrolides were 44.6% and 40.5%, respectively (Table S2).

The number of CAP cases more than doubled from 67 in 2012–2013 to 143 in 2014–2015. CAP was treated with antibiotics in 89.6% and 86.8% of cases, respectively (Fig. 2b). During the post-guideline period, antibiotics were prescribed less often in private (56.3%) than in public primary care clinics (84.6%; *p* = 0.037) and in PED (94.3%; *p* < 0.001 vs. private and *p* = 0.091 vs. public primary clinics) (Fig. 2b).

The number of non-wheezing bronchitis cases was 312 in 2012–2013 and 355 in 2014–2015. Non-wheezing bronchitis was treated with antibiotics in 77.6% and 71.0% of cases, respectively (Fig. 2c). During the post-guideline periods, antibiotics were prescribed more often in private (80.9%) than in public primary care clinics (58.8%; *p* < 0.001) or PED (11.1%; *p* < 0.001 vs. private and *p* = 0.011 vs. public primary) (Fig. 2c).

The number of wheezing bronchitis cases was 264 in 2012–2013 and 290 in 2014–2015. Wheezing bronchitis was treated with antibiotics in 4.2% and 2.8% of cases, respectively. During the post-guideline periods, antibiotic prescription rate was 15.8% in private and 4.4% in public primary care (*p* = 0.115) and 1.0% in PED (*p* = 0.005 vs. private and 0.103 vs. public primary) (Fig. 2d).

### Guideline concordance

The Current Care Guidelines do not recommend antibiotics for bronchitis. Thus, among children with non-wheezing bronchitis, only those 29.0% who did not receive antibiotics were treated in concordance with guidelines. The concordance figures were 10.7% and 41.2% in private and public primary care, respectively. For wheezing bronchitis, the overall guideline concordance was 97.2%. For CAP, the Current Care Guidelines recommend antibiotics, and amoxicillin is the guideline-concordant choice at all ages. Amoxicillin can be combined with macrolides in > 5-year-olds or with doxycycline in > 8-year-olds, if atypical bacteria are suspected. By these criteria, the guideline concordance was 65.9% in PED and 59.0% in public and 50.0% in private primary care clinics (Table 1).

**Table 1:** Guideline concordance of antibiotic prescriptions for community-acquired pneumonia during the post-guideline seasons presented separately for the pediatric emergency department and public and private primary care clinics. P-values to compare differences between providers

Guideline concordance	Public primary care clinics^1^, *n* = 39	Pediatric emergency department^2^, *n* = 88	Private primary care clinics^3^, *n* = 16	*p* value		
No^a^	6 (15.4%)	5 (5.7%)	7 (43.8%)	0.091 (^1^vs.^2^)	0.037 (^1^vs.^3^)	<0.001 (^2^vs.^3^)
Yes^b^	23 (59.0%)	58 (65.9%)	8 (50.0%)	0.453 (^1^vs.^2^)	0.542 (^1^vs.^3^)	0.224 (^2^vs.^3^)
Yes^c^	25 (64.1%)	63 (71.6%)	8 (50.0%)	0.399 (^1^vs.^2^)	0.322 (^1^vs.^3^)	0.088 (^2^vs.^3^)

^a^No antibiotics

^b^Amoxicillin monotherapy or amoxicillin + macrolide at all ages or amoxicillin–doxicycline in > 8-year-olds

^c^In addition to b, macrolide monotherapy accepted in > 5-year-olds and doxycycline monotherapy accepted in > 8-year-olds

## Discussion

The current study had four main results regarding pre- and post-guideline antibiotic prescriptions for LRTI in three settings of pediatric health care. First, the overall antibiotic prescription rates were similar (48%) during the pre- and post-guideline study periods. The rate was lowest (25%) in public primary, next (46%) in PED, and highest in private (75%) primary care clinics. Second, the antibiotic prescription rates for non-wheezing bronchitis did not change after the guideline release but differed by site. During the post-guideline study periods, the rate was highest in private (81%) followed by public primary care clinics (59%) and PED (11%). Third, macrolide prescriptions were common. During the post-guideline study periods, the proportion of macrolide prescription was highest in private (41%) followed by public primary care clinics (25%) and PED (1%). Fourth, the rates of antibiotic prescription for CAP, which is recommended by the guidelines, did not change after the guideline release but again differed by site. During the post-guideline study periods, the rate was highest in PED (94%), followed by public (85%) and private (56%) primary care clinics.

Another Finnish study included 89,359 children who visited private primary care clinics for LRTI in 2014–2020 and found a gradual decrease in antibiotic prescriptions from 37% in 2014 to 20% in 2020 and in macrolide prescriptions from 17 to 8%, respectively [8]. That study [8] suggested that the 2014 guidelines had an impact on antibiotic prescriptions, but the change was slow. In line with our results, CAP was undertreated and bronchitis was overtreated with antibiotics. Slow effects of evidence-based guidelines for children’s respiratory infections have been reported from primary care clinics in the USA [9–11] and from emergency departments in France [12–15]. Macrolide overuse was common in the current study, as was in an American observational study on CAP in children treated at home [16]. We have previously reported two beneficial and immediate changes that occurred after the guideline release in 2014 [17, 18]. The use of nebulized racemic adrenaline for bronchiolitis decreased in Finnish children’s hospitals [17], and the numbers of chest radiographs in children decreased in the national register data [18].

Less than 15% of children in the current study had CAP. The 87% antibiotic prescription rate is in line with those in previous studies on CAP in children [12, 15, 19, 20] as well as with the Finnish [4] and international guidelines [2, 3]. All CAP patients, except young children with mild symptoms, should be treated with antibiotics. An American nationwide ambulatory care sample included 348 CAP cases, and the antibiotic prescription rate was 79% [19]. In a French nationwide emergency department network, the prescription rates for CAP in children varied between 83 and 86% by season [12], increased to 99% after guideline implementation [20], but varied by doctoral specialty between 80 and 99% [15]. In the Netherlands, the prescription rate was 67–72% among 652–1515 visits for pediatric CAP in 2010–2012 [21].

There is wide consensus that bronchitis is self-improving viral infection, and the current guidelines, including the Finnish Current Care Guidelines, advise to refrain from prescribing antibiotics [4, 5]. A prospective randomized placebo-controlled double-blinded study from England showed that the effect of amoxicillin was not superior to that of placebo in 400 children aged 0.5–12 years with uncomplicated LRTI [22]. In the current study, antibiotics were prescribed for 59% and 81% of non-wheezing bronchitis cases in public and private primary care clinics, respectively. Although unnecessary, the rates are within the ranges published in other countries. A Japanese register study reported antibiotic prescription rates of 45% and 69% for 1360 and 1118 visits due to bronchitis in children aged < 10 and > 10 years, respectively [23]. In the USA, bronchitis was treated with antibiotics in 55% of 491 visits [19], and in the Netherlands, in 44–47% of 1510–2813 visits [21]. The results of the current and previous studies indicate that antibiotic prescription rates for bronchitis need to be lowered, and indeed, in the latest study in Finland, the prescription rate was only 20% in 5050 children with uncomplicated bronchitis in 2020 [8].

### Concordance

Antibiotic avoidance was the guideline-concordant treatment in wheezing and non-wheezing bronchitis, and in primary care, the post-guideline concordances were 45–100% for wheezing bronchitis but only 20–30% for non-wheezing bronchitis. When the strict criteria (either amoxicillin as monotherapy or amoxicillin and macrolides or doxycycline combined) were applied for CAP, 50–65% of the prescriptions were guideline-concordant in primary care and nearly 72% in PED. The use of macrolide monotherapy, even though atypical bacteria are suspected, is against the guidelines. However, prescribing of two antibiotics is impractical and not superior to beta-lactam monotherapy, as was shown in 1418 American children hospitalized for CAP [24]. In the present study, adherence to guidelines was higher in public than in private sector and at the secondary than primary healthcare level by all measures.

### Implementation

The slow and limited effect of evidence-based guidelines for respiratory tract infections in children has been revealed in studies conducted in primary care clinics [9–11] and emergency departments [12–15]. In addition to release, more active implementation processes are needed to increase and accelerate the influence of evidence-based guidelines. In a Finnish private healthcare service company, as high as 89% reduction in prescriptions of antitussives for children was achieved by a company-level intervention consisting of instructions, educational meetings, clinic visits, and targeted personal feedback [25]. In the USA, a 6-month personalized audit and feedback program including 102 attendants and 214 controls was carried out in primary care clinics [26] and the guideline-concordant prescriptions for CAP were more frequent (94%) in the intervention than in the control group (79%) [26]. In another American study, the 12-month intervention consisted of lectures and personal audits, and prescribing of broad-spectrum antibiotics for respiratory infections decreased from 27 to 14%, but relapsed to 28% after intervention [9].

The factors that contribute to physicians’ non-adherence to guidelines ought to be more comprehensively understood. In a qualitative questionnaire-based study conducted in Ireland, GPs cited the following reasons for their non-compliance: they perceived the guidelines as not being comprehensive for real-life scenarios, experienced pressure from patients or parents, and were influenced by their own perceptions of patient expectations [27].

### Strengths and limitations

The design of the current study, which allowed comparisons of three levels of healthcare, and of pre-guideline and post-guideline periods, is undoubtedly a strength. The retrospective nature is a limitation but, on the other hand, means that clinicians were not aware of the study, and the study did not affect their clinical choices. The data on the post-guideline period were collected from years 2014–2015. Since then, a decreasing trend in prescribing for bronchitis has been observed but overprescribing is still common [8].

There were differences in patients between the public and private sectors and especially between the primary and secondary healthcare levels. The different distributions of diagnoses weakened the comparisons between different sites and levels. Also, we did not know previous antibiotic treatments and antibiotic allergies, which might have affected antibiotic choices. Since the collected cases were coded separately for each site, some overlapping between the groups probably existed but was not possible to take into account in analyses.

## Conclusion

The current study found no immediate impact of the publication of the Finnish Current Care Guidelines for children’s LRTIs in 2014. The antibiotic prescriptions remained similar for the infection seasons before and after the guideline release. However, we observed differences in antibiotic prescriptions between public and private sectors and between primary and secondary healthcare levels. The high number of antibiotic prescriptions for bronchitis and wide use of macrolides in private primary care raise concerns and calls for more active implementation of guidelines.

### Supplementary Information

Below is the link to the electronic supplementary material.

Supplementary file1 (DOCX 22 kb)

Supplementary file2 (DOCX 22 kb)

## Data Availability

In accordance with privacy and data protection guidelines, the raw data supporting the conclusions of this article will not be made available publicly to ensure patient confidentiality. Researchers interested in the data for academic purposes may contact the corresponding author, with each request being subject to an ethical and legal review.

## Abbreviations

CAP: Community-acquired pneumonia
CI: Confidence interval
ICD: International Classification of Diseases
LRTI: Lower respiratory tract infection
