# Supplementary material for: Use of antibiotics contrary to guidelines for children’s lower respiratory tract infections in different health care settings
Source: Eur J Pediatr. 2023 Jul 19;182(10):4369–77. doi: 10.1007/s00431-023-05099-6 (PMC10587298; doi:10.1007/s00431-023-05099-6)
Supplement: Supplementary file 1 — Supplementary file1 (DOCX 22 kb) [file 431_2023_5099_MOESM1_ESM.docx]

| Table S1. Basic characteristics of study group shown separately for different healthcare providers. | | | | | | | |  |
| --- | --- | --- | --- | --- | --- | --- | --- | --- |
|  | **Age, median (Q1, Q3)** | | | **Girls, %** | | | |  |
|  | Before | After | p-value | | Before | After | p-value | |
| Public primary care clinics | 2.6 (1.3, 5.9) | 3.8 (1.7, 8.8) | 0.060 | | 40.1 | 39.6 | 0.913 | |
| Pediatric Emergency Department | 1.3 (0.6, 2.5) | 1.7 (0.7, 3.2) | 0.020 | | 37.6 | 34.9 | 0.522 | |
| Private primary care clinics | 4.5 (2.0, 8.9) | 5.3 ( 2.6, 9.4) | 0.136 | | 43.8 | 41.7 | 0.618 | |
| All | 2.5 (1.2, 5.8) | 3.1 ( 1.3, 7.3) | 0.050 | | 40.8 | 38.5 | 0.372 | |
