# Supplementary material for: Use of antibiotics contrary to guidelines for children’s lower respiratory tract infections in different health care settings
Source: Eur J Pediatr. 2023 Jul 19;182(10):4369–77. doi: 10.1007/s00431-023-05099-6 (PMC10587298; doi:10.1007/s00431-023-05099-6)
Supplement: Supplementary file 2 — Supplementary file2 (DOCX 22 kb) [file 431_2023_5099_MOESM2_ESM.docx]

| Table S2. Antibiotic prescriptions for 699 children during the pre- and post-guideline study periods, presented separately for the Pediatric Emergency Department and public and private primary care clinics | | | |
| --- | --- | --- | --- |
| Antibiotics | **2012–2013, pre-guideline, n (%)** | **2014–2015, post-guideline, n (%)** | **All, n (%)** |
| Public primary care clinics | 73 | 113 | 186 |
| Amoxicillin | 39 (53.4%) | 65 (57.5%) | 104 (55.9%) |
| Amoxicillin + clavulanic acid | 5 (6.8%) | 9 (8.0%) | 14 (7.5%) |
| Azithromycin | 18 (24.7%) | 23 (20.4%) | 41 (22.0%) |
| Other macrolides | 6 (8.2%) | 6 (5.3%) | 12 (6.5) |
| Pediatric Emergency Department | 43 | 86 | 129 |
| Amoxicillin | 33 (76.7%) | 60 (69.8%) | 93 (72.1%) |
| Amoxicillin + clavulanic acid | 8 (18.6%) | 14 (16.3%) | 22 (17.1%) |
| Azithromycin | 0 | 1 (1.2%) | 1 (0.8%) |
| Other macrolides | 0 | 0 | 0 |
| Private primary care clinics | 197 | 186 | 383 |
| Amoxicillin | 56 (28.4%) | 65 (34.9%) | 121 (31.6%) |
| Amoxicillin + clavulanic acid | 24 (12.2%) | 31 (16.7%) | 55 (14.4%) |
| Azithromycin | 69 (35.0%) | 65 (34.9%) | 134 (35.0%) |
| Other macrolides | 19 (9.6%) | 10 (5.5%) | 29 (7.6%) |
| All | 313 | 385 | 698 |
| Amoxicillin | 128 (40.9%) | 190 (49.4%) | 318 (45.6%) |
| Amoxicillin + clavulanic acid | 37 (11.8%) | 54 (14.0%) | 91 (13.0%) |
| Azithromycin | 87 (27.8%) | 89 (23.1%) | 176 (25.2%) |
| Other macrolides | 25 (8.0%) | 16 (4.2%) | 41 (5.9%) |
